# Logistics management information system performance for program drugs in public health facilities of East Wollega Zone, Oromia regional state, Ethiopia

**DOI:** 10.1186/s12911-018-0720-9

**Published:** 2018-12-17

**Authors:** Kefyalewu Tiye, Tadesse Gudeta

**Affiliations:** 1 Nekemte Referral Hospital, East Wollega Zone, Oromia regional state Ethiopia; 2 Pharmaceutical supply chain management specialist, School of Pharmacy, Faculty of Health Sciences, Jimma University, Jimma, Ethiopia

**Keywords:** Logistics management information system, Health facility, Performance, Program drugs, Ethiopia

## Abstract

**Background:**

Proper logistics management information system in the supply chain improves health outcomes by maintaining accurate and timely information. The purpose of this study was to determine program drugs logistics management information system performance in public health facilities of East Wollega Zone, Oromia Regional State.

**Methods:**

A facility-based descriptive cross-sectional study design complemented with a qualitative method was conducted from April 01 to May 30, 2017. The quantitative data were gathered through reviewing logistics tools, a physical count of the selected program drugs, and interview of the pharmacy staffs. The evaluation of data quality was done on 134 RRFs and 805 bin-cards. A statistical package for social science version 20 was used to analyze the quantitative data. A chi-square test was performed to determine the association between dependent and independent variables. For the qualitative method, 11 face to face in-depth interviews were carried out, and the data were analyzed using thematic analysis technique.

**Results:**

Twenty three selected public health facilities were included in the study of which 39% of them had an automated recording system. Concerning the data quality, 65% of RRFs and 79.1% of bin-cards were accurately filled, and 97.8% of the reports were found to be complete. Sixty-nine percent of the facilities had timely submitted their report to the higher level and the reporting rate of the facilities was determined to be 97%. A significant association was observed between RRF data accuracy and type of profession, *X*^*2*^ (4, *N = 134*) =35.0, *P* = 0.040, trainings, *X*^*2*^ (2, *N* = 134) =37.12, *P* = 0.001, e-LMIS, *X*^*2*^ (2, *N = 134*) = 38.67, *P* = 0.03, educational status, *X*^*2*^ (2,*N* = 134) = 90.38, *P* = 0.012, & supervision, *X*^*2*^ (2, *N* = 134) = 94.03,*P* < 0.001. Shortage of skilled human resources and poor commitment of the staffs were identified to be the major bottlenecks of logistics management information system performances.

**Conclusions:**

The facilities’ report submission rates were promising yet the quality of the reports need improvement. Poor data quality was more likely because of weak supportive supervision and the information system being managed by non-pharmacy professionals.

## Background

A quality health service requires the availability of safe, effective, affordable and qualified drugs in adequate quantity at all times with appropriate dose and dosage forms. However, managing drug supply is a very complex process that requires a strong organizational structure, and integrated supply chain [1]. It involves a number of interrelated logistics functions accompanied by appropriate support functions in a supply chain and governed by stringent policy and legal framework [2]. These functions can be kept effective and integrated well if quality information moves up and downstream of a supply chain [3]. Thus, a properly managed information systems should be established in each health supply chain facilities.

Logistics management information system (LMIS) across all the supply chain levels increases program impacts, i.e. maintains commodity availability and improves service seeking of the community, enhances the quality of care, increases professional satisfaction and morale. Motivated staff are more likely to deliver a higher quality of service, improves efficiency and effectiveness [4].

A logistics management information system is a subset of organizations total information systems. It involves records and reports used to gather, analyze, and validate data from all levels of the logistics system that can be used to make logistics decisions and manage the supply chain [5, 6]. Therefore, logistics records are important parts of every logistics system. The records are intended to gather & record vital logistics data at each level of the health care system. The data are then combined to form logistics reports, which are used for critical decision-making about resupply quantities, forecasting, and procurement decisions [7].

Based on the actions taken on supplies in the logistics system (i.e. storing, moving and using the supplies), three basic types of logistics records are used to monitor and track the status of the products in the pipeline. It includes stock keeping records, transaction records, and consumption records. Stock keeping records hold information about products in a storage e.g. bin-cards and stock-cards whereas the transaction records keep information about products being moved e.g. report & resupply forms (RRFs), internal facility & resupply forms (IFRRs), different vouchers & etc. The consumption records maintain information about products being consumed at the health facility e.g. patient registration book [8, 9].

The quality and timeliness of the LMIS records and reports have a sound impact on the sustainable accessibility of essential medicines [10]. Supply chain facilities including service delivery points (SDP) should maintain the data quality of their LMIS tools and also submit their reports within the specified schedule, especially for program drugs like HIV, TB, malaria, maternal and child health (MCH) and family planning products [1]. The first three problems are the major global public health threats and cause substantial morbidity, mortality, negative socio-economic impacts, and human suffering. MCH and family planning are also the major challenges in low-income countries next to HIV, TB, and malaria [11, 12]. Products for these problems are not easily accessible in developing countries; they are obtained through the financial aid of non-governmental organizations from international suppliers [13]. Therefore, to minimize the procurement lead times and unnecessary costs, quality and timely information is mandatory [14]. Lack of access to essential health products in the majority of developing countries, particularly in sub-Saharan Africa is mostly due to poor LMIS data quality and absence of real-time information [15]. For instance, a study conducted in Tanzania in 2006 indicated, data for logistic decision-making in the public health system was largely unavailable, resulting in the procurement and distribution decisions based on incomplete information. Even, there was a poor integration of the different logistics functional processes, from procurement to inventory management which led to inefficiencies and minimal logistics data visibility within a central medical store and in zones and health facilities [16]. A cross-sectional study conducted in Malawi also indicated that lack of integrated information systems and poor data quality in the majority of public health facilities have resulted in poor data visibility and difficulty in decision making [6]. In 2012, systems for improved access to pharmaceuticals and services (SIAPS) conducted an assessment on implementation and use of LMIS in Mali and identified a number of problems including inaccessibility of data for supply chain managers, weak reporting systems, as well as the follow of inaccurate and untimely information in the supply chain system of the country [17]. A similar study in Lesotho showed that incorrect recording of data on stock cards & reports, delayed report submission, lack of uniform and simple data collection tools made logistics decision very challenging which in turn led to frequent stock-outs of medicines and health commodities [18].

An information system fails because of different factors and challenges including chronic stock outs, poor supervision, and monitoring, the absence of regular reporting of stock level and consumption trends, lack of top management support [19]. A study conducted in Ghana also revealed that shortage of health service providers, inadequate logistics recording & reporting tools, and lack of appropriate essential data from the service delivery points, lack of commitments are some of the major challenges negatively affecting logistics management in the healthcare facilities [20].

In Ethiopia, studies on logistics management information system are very limited and even most of them are simply surveys reporting a description of the LMIS operating across the health facilities. Therefore, the aim of this study was to evaluate the logistics management information system performance for program drugs and also explore the associated challenges in the public health facilities of East Wollega Zone, Oromia regional state, Ethiopia.

## Methods

### Study area

The study was conducted in selected public health facilities of East Wollega zone, Oromia regional state, Western Ethiopia. East Wollega zone covers a land mass of 13,820.23 km^2^ that is 3.97% of Oromia land coverage. It is administratively sub-divided into seventeen districts. The zone is delivering health services to the community with three hospitals, sixty-one health centers (one NGO) and 294 health posts. It has 1093 different health professionals (i.e.106 pharmacy, 677 health extension workers, and 395 administrative workers) [source, East Wollega Zonal health office].

### Study design and period

The study employed a facility based cross-sectional descriptive study design complemented with a qualitative study to evaluate the LMIS performance of the facilities including data quality, reporting rates, and the associated challenges. The study was conducted from April 01 to May 30, 2017.

### Study population and sampling procedures

**Fig. 1:**
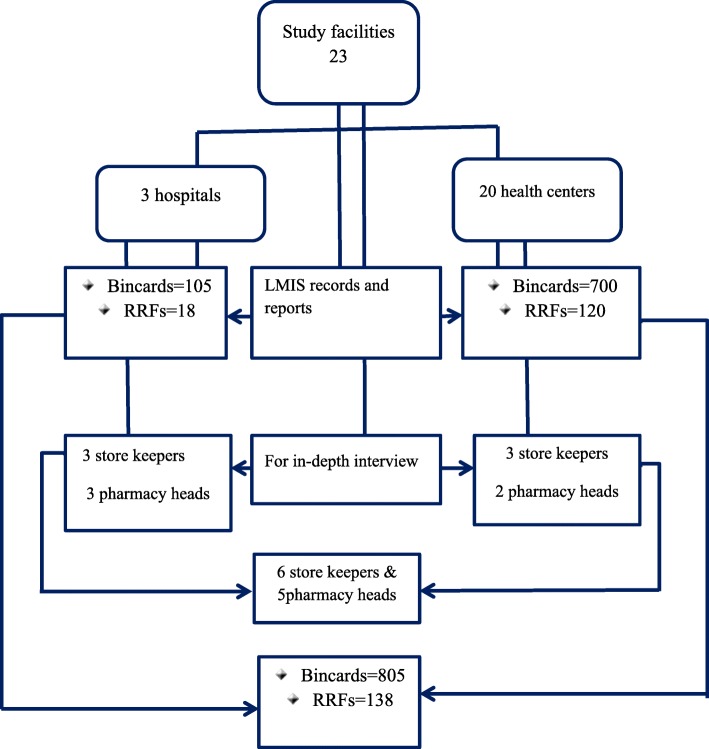
Flow diagram showing documents and personnel selection for the study

Twenty-three public health facilities, i.e., three public hospitals and 20 health centers were included in the study. Logistics records and reports from each facility were reviewed to evaluate data quality and reporting rates. The recording and reporting tools were proportionally selected from each facility. The sampling process of the elements (i.e.documents & personnel) is shown in the diagram below (Fig. 1). The sample size of the health facilities was determined based on the USAID delivery project guideline (i.e., a logistics indicators assessment tool) that recommends taking at least 15% of the total facilities to increase the power of generalizability [21]. Accordingly, twenty-three health facilities were taken from a total of 63 public health facilities. The health centers were clustered based on their geographical distribution (districts). Those health centers which manage common program drugs were conveniently selected from each cluster until the required sample size was achieved. The hospitals were included by default since they are situated in different districts and also manage all the program drugs. Regarding documents, two LMIS tools (report and requisition forms (RRFs) and bin-cards) were reviewed to evaluate data quality since they are the most commonly used tools to record and transfer data in the public health facilities in Ethiopia. According to the integrated pharmaceutical logistics system (IPLS) of Ethiopia, public health facilities (hospitals and health centers) are expected to submit their RRFs to higher level bimonthly [8]. Accordingly, the report and requisition forms of one year, i.e., six RRFs from each health facility were planned for review. Unfortunately, four of the health facilities submitted only five of the expected total reports. Thus, 134 RRFs were reviewed to evaluate the data quality of the reports.

The data accuracy of RRF was evaluated by computing the discrepancy between the stock record balance on the bin-cards and RRFs. For this purpose, the date and document number of the transactions were checked to pick the corresponding bin-cards. The other type of LMIS tool reviewed for data accuracy was bin-card. The sample size of the bin-cards was determined based on the number of products included in the study. Sixty specific program drugs were managed by the health facilities of which 35 were common to the study facilities [Source, East Wollega zonal health office]. Therefore, 35 bin-cards from each health facility were reviewed to determine LMIS record accuracy. The study also assessed the availability and utilization of other logistics tools like internal facility report and requisition form (IFRR). IFRR is a document used by dispensaries within the health facilities to report drug consumption to a medical store and request quantities for the next consumption.

### Measurements of LMIS data quality and facility reporting rates

The data quality of LMIS was measured using indicators such as accuracy, completeness, and timeliness.

1. The data record accuracy measures whether stock balances on bin-cards are similar to the actual inventory on hand.

This can be determined using the following formula:

$$ \mathrm{Record}\ \mathrm{accuracy}=\frac{\Big(\mathrm{Ending}\ \mathrm{balance}\ \mathrm{on}\ \mathrm{bin} \operatorname {card}-\mathrm{physical}\ \mathrm{stock}\ \mathrm{counts}}{\mathrm{physical}\ \mathrm{stock}\ \mathrm{counts}}\ast 100 $$

2) Accuracy in transferring data from records (e.g. bin-cards) to the LMIS reporting forms (e.g. RRFs) provides information on how well the health facilities are accurately tracking their inventories and transferring this information to the LMIS report forms. It is determined using the following formula;

$$ \mathrm{Data}\ \mathrm{transfer}\ \mathrm{accuracy}=\frac{\Big(\mathrm{stock}\ \mathrm{balance}\ \mathrm{on}\ \mathrm{RRFs}-\mathrm{Ending}\ \mathrm{balance}\ \mathrm{on}\ \mathrm{bin}-\mathrm{cards}\ }{\mathrm{Ending}\ \mathrm{balance}\ \mathrm{on}\ \mathrm{bin}-\mathrm{cards}} \ast 100 $$

3) The completeness of reports: Data from five major program drugs (i.e. HIV, opportunistic infection (OI), TB, malaria, and family planning included in the RRF) were used to check the completeness of the reports. A report is considered complete if all the columns for each product listed in the report are filled in for at least one product listed under each program unless the facility does not manage the product [22].
4) Timeliness: As per the integrated pharmaceutical logistics system (IPLS) of Ethiopia, A report is said to be timely submitted if,

- The health centers directly submit their RRF to the higher supplier (PFSA) until the 10th day after the reporting period or if they submit to woreda health office until the 5th day after the reporting period and
- The hospitals submit their reports to pharmaceutical fund and supply agency (PFSA) or regional health bureau until the 10th day after the reporting period [8]

5) Facility reporting rates: The facility reporting rate was computed using the following formula (2).

$$ \mathrm{Reporting}\ \mathrm{rate}=\frac{\left(\mathrm{number}\ \mathrm{of}\ \mathrm{facilities}\ \mathrm{submit}\mathrm{ted}\ \mathrm{the}\mathrm{ir}\ \mathrm{report}\ \mathrm{in}\ \mathrm{the}\ \mathrm{specified}\ \mathrm{schedule}\right)\ }{\mathrm{a}\ \mathrm{total}\ \mathrm{number}\ \mathrm{of}\ \mathrm{facilities}\ \mathrm{expected}\ \mathrm{to}\ \mathrm{submit}\ \mathrm{the}\mathrm{ir}\ \mathrm{report}}\ast 100 $$

### Data collection procedures

Semi-structured questionnaires and checklists adapted from USAID deliver guidelines were used to collect the necessary data. Socio-demographic data, facilities’ reporting rates, availability and utilization of reporting and recording formats, data quality of RRF & bin-cards and factors associated with LMIS performance were collected. The data were gathered through a physical count of drugs, review of relevant documents, and interview of the health facility store managers and pharmacy heads. Regarding document review, logistic reports of one year and recording tools (bin-cards) on the day of the visit were evaluated.

### Data processing & analysis

A statistical package for social sciences (SPSS) version 20 was used to encode and analyze data. Descriptive and inferential statistical analyses were done and the outputs were then summarized using tables & figures. A chi-square test was run to determine the associations between the dependent and independent variables. A critical values *p* < 0.05 were considered as statistically significant.

### For the qualitative study

Eleven face to face in-depth interviews were conducted to collect qualitative data. The participants had experience of greater than 5 years in health logistics management. Currently, storekeepers and pharmacy heads of the public health facilities in Ethiopia play the lion’s share of logistics management practice. Therefore, six storekeepers and five pharmacy heads were selected based on their service experience. The interviews were conducted by one of the researchers to maintain the consistency of information.Written informed consent was obtained from the participants to audiotape the interview. The interviews were conducted in a local language Afan Oromo, and it was tape recorded. The participants were asked in-depth and probing questions to get broad information on the topic of interest. Each interview on average took 20 min. The record was then transcribed to English language by the researchers and verified by two experts from Jimma University academic staffs. Then, the findings were analyzed manually using the thematic analysis technique. Accordingly, the authors familiarized themselves with the data by taking notes of the interview. The data were then coded by using a table in a word document. The coded data were organized to search for themes. The final step was naming and describing the themes and producing the report.

### Data quality assurance

A pilot study was conducted in 5% of the facilities to check the data collection tools for its contents and any ambiguous questions. The facilities involved in pretest were excluded from the actual study. The data collectors were also trained for 30 min on how to complete the tools. On top of that, the principal investigators closely supervised the data collectors. Each time after data collection, the questionnaires were checked for completeness.

### Operational definitions

- **Bin-card accuracy:** is a measure of a discrepancy between the stock balance on bin-cards and physical count of the products
- **RRFs data accuracy:** is a measure of a discrepancy between the stock balance on RRFs and ending balance on bin-cards. Accordingly, the record/report is said to be accurate if zero discrepancies are calculated and near accurate if the discrepancy is ±10% and non-accurate if the difference is > ±10% [22]. However, for the purpose of the chi-square test, the near accurate outcome variable was merged to not accurate
- **Completeness:** A report is considered complete if all the columns for each product listed in the report are filled in for at least one product listed under each program unless the facility does not manage the product.
- **Data quality**: it is the accuracy, completeness, and timeliness of logistics data.
- **Program drugs:** in the current study are drugs used to treat or prevent TB/ leprosy, HIV/AIDS, malaria, maternal & child health (MCH) & family planning used both in health centers and hospitals.
- **Pharmacy professionals:** in the current study include pharmacists (degree holders), and pharmacy technicians (diploma holders)
- **Timelines:** the reporting schedule in which the facilities are expected to submit their reports (RRFs) to the next higher officials. According to IPLS of Ethiopia [8], the report is said to be submitted timely if
  - ○ The hospitals submit their reports to pharmaceutical fund and supply agency (PFSA) or regional health bureau until the 10th day after the reporting period, and
  - ○ The health centers directly submit their RRF to the higher supplier (PFSA) until the 10th day after the reporting period or if they submit to woreda health office until the 5th day after the reporting period.
- **Training**: training in this study refers to a short-term supply chain training which might improve the logistics information system, e.g.integrated pharmaceutical logistics system (IPLS) training.
- **Service year:** The pharmacy staffs service experience on logistics management.
- **Feedback report:** Is a type of report used by the suppliers to inform lower level facilities about their performance
- **Supervision:** An activity carried out by the top level management of the health facilities’ and higher officials outside the facilities (e.g.District/Woreda health office and zonal health office) to oversee the logistics management practice of the facilities.

## Results

### The socio-demographic characteristics of staffs

Sixty-one staff of different professions were working under pharmacy units of the facilities, of which 37 (60.7%) were pharmacy professionals. The majority of the staff, 57 (93.4%) were male. Regarding educational qualification, 30 (49.2%) of the staff were degree holders, and the remaining 31 (50.8%) were diploma qualifiers. Forty-one (67.2%) of the staff had service year of less than 5. More than half, 33 (54.1%) of the staff were working in the dispensary and 23 (37.7%) were serving as storekeepers (Table 1).

**Table 1:** The profile of the staffs working under pharmacy units of the public health facilities in the East Wollega zone, May 30/2017 (hospitals = 3, health centers = 20)

Socio-demographic variables		Types of facilities		Total (%)
		Hospital (%)	Health center (%)	
Gender	Male	25 (92.6)	32 (94.1)	57 (93.4)
	Female	2 (7.4)	2 (5.9)	4 (6.6)
	Total	27 (100)	34 (100)	61 (100)
Educational qualification	Degree	25 (92.6)	5 (14.7)	30 (49.2)
	Diploma	2 (7.4)	29 (85.3)	31 (50.8)
	Total	27 (100)	34 (100)	61 (100)
Type of profession	Pharmacy	27 (100)	10 (29.4)	37 (60.7)
	Nurse	0	24 (70.6)	24 (39.3)
	Total	27 (100)	34 (100)	61 (100)
Service year	≤5 year	20 (74)	21 (61.8)	41 (67.2)
	> 5 year	7 (26)	13 (38.2)	20 (32.8)
	Total	27 (100)	34 (100)	61 (100)
Position	Dispensers	19 (70.4)	14 (41.2)	33 (54.1)
	Storekeepers	5 (18.5)	18 (52.9)	23 (37.7%)
	Pharmacy head	3 (11.1)	2 (5.9)	5 (8.2)
	Total	27 (100)	34 (100)	61 (100)

### Staffs empowerment

#### Integrated pharmaceutical logistics system /LMIS training

**Fig. 2:**
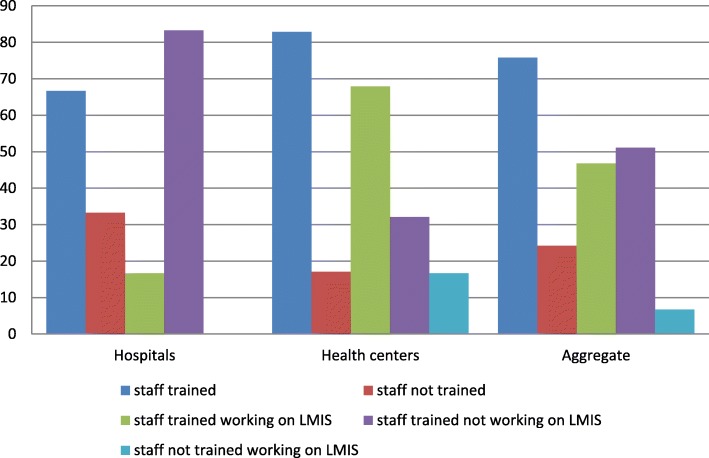
Staff training on IPLS/LMIS & their current practice in selected public health facilities of East Wollega zone, May 30/2017 (Hospitals = 3, health centers = 20)

From the total of 61 pharmacy staff in the study facilities, 47 (75.8%) had received IPLS/ LMIS training. Per type of facility,18 (66.7%) of the hospitals’ staff and 28 (82.9%) of the health centers’ staff had received the training. Of the trained staff, 3 (16.7%) of the hospitals’ and 19 (67.9%) of the health centers’ staff were working on LMIS management (Fig. 2).

#### Supervision and feedback support from top level management

**Fig. 3:**
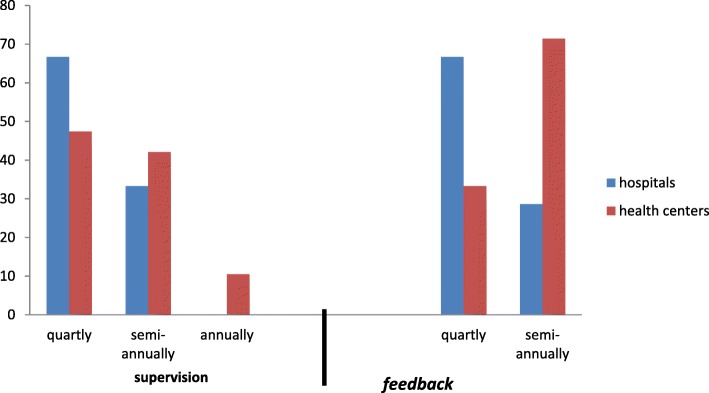
The frequency of supervision & feedback report from the higher level management in selected public health facilities of East Wollega zone, May 30/2017 (Hospitals = 3, health centers = 20)

Twenty-two (95.56%) of the study facilities had received supportive supervision, and 10 (43.48%) of the facilities had received a feedback report. Among the facilities that received supportive supervision, 11 (50%) of them received quarterly, and only 2 (9%) of them received annually. Based on the type of facility, 19 (95%) of the health centers and all of the hospitals had received the supervision. Two (67%) of the hospitals had got supervision quarterly, and the health centers had also received quarterly, 9 (47.4%) and semi-annually, 8 (42.1%). Seven (35%) of the health centers and all of the hospitals had received feedback from the higher level. Of the facilities that had received feedback, 6 (60%) of them received semiannually, and 4 (40%) of them received quarterly. However, the disaggregated data revealed that 2 (66.7%) of the hospitals and 5 (71.4%) of the health centers had received feedback quarterly and semi-annually respectively (Fig. 3).

### Availability of LMIS tools & its utilization

All of the study facilities had a manual LMIS reporting and recording formats, and also used them to capture logistics data. Regarding e-LMIS, all of the hospitals had an automated recording and reporting system, but it was functional in 2/3 (66.7%) of the hospitals. However, only 6 (30%) of the health centers had an automated LMIS (Table 2).

**Table 2:** Availability and utilization of LMIS reporting and recording formats in selected public health facilities of East Wollega zone, May30/2017. (Hospitals = 3, health centers = 20)

LMIS* report & recording formats, availability & utilization		Hospitals % ages	Health centers % ages	Aggregate % ages
Bin card	Available	100	100	100
	Utilized	100	100	100
RRF**	Available	100	100	100
	Utilized	100	100	100
IFRR***	Available	100	100	100
	Utilized	100	65	69.6
Computer	Available	100	30	39
	Utilized	66.7	30	34.8

^*^Logistics management information system,^**^Report and resupply form, ^***^Internal facility report and resupply form

### Data quality of LMIS report and records

#### RRF data quality

**Fig. 4:**
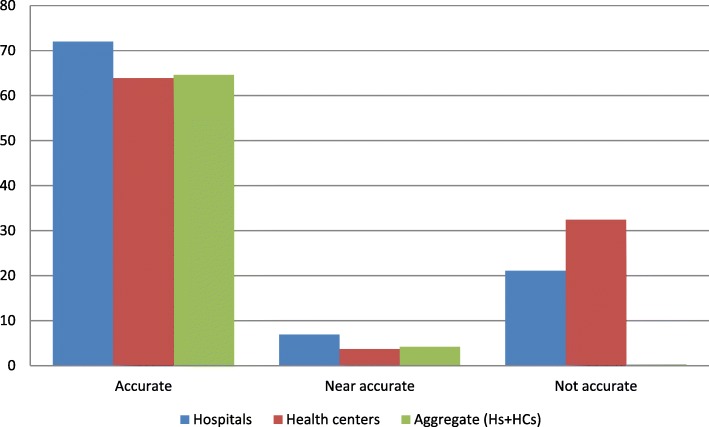
The accuracy of RRF data by facility type and the overall aggregate performance of selected public health facilities in East Wollega zone, May 30/2017

The data quality of RRF was assessed using indicators such as accuracy, timeliness, and completeness. Accordingly, of the total sampled RRF, 89 (64.6%) of them were accurately filled, and 43 (31.2%) were found to be inaccurate. However, when we consider by facility type, the hospitals and the health centers respectively filled 13 (72%) and 77 (63.9%) of their RRFs accurately (Fig. 4).

From inferential statistical analysis, RRF data accuracy had a significant association with staff training, *X*^*2*^ (2, *N* = 134) =37.12, *P* = 0.001, level of education, *X*^*2*^ (2,*N* = 134) = 90.38, *P* = 0.012, type of profession, *X*^*2*^ (4, *N = 134*) = 35.0,*P* = 0.040, availability of electronic bin-cards, *X*^*2*^ (2, *N = 134*) = 38.67,*P* = 0.030, supervision, *X*^*2*^ (2, *N* = 134) =94.03, *P* < 0.001 and feedback *X*^*2*^ (2, *N* = 134) = 21.68, *P* = 0.045 (Table 3).

**Table 3:** The association of RRF data accuracy, and possible contributing factors in the selected public health facilities of East Wollega zone, May30/2017

Variables	RRF* data accuracy		
	Pearson chi-square		
	Value	Df**	*P*-values
Service year	.465	2	0.793
Training	37.115	2	0.001
Availability of electronic bin-card	38.666	2	0.030
Educational status	90.384	2	0.012
Type of profession	34.999	4	0.040
Supervision	94.029	2	0.000
Feedback	21.684	2	0.045

* Report and resupply form,**degree of freedom

### Report submission rates, timeliness, and completeness of the reports

**Fig. 5:**
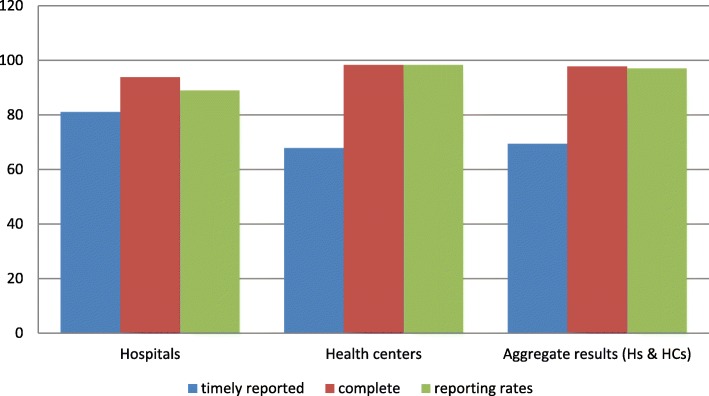
Distribution of facilities’ reporting rates & timelines and completeness of RRFs in selected public health facilities of East Wollega zone, May30/2017

Regarding reporting performance, the study facilities had a reporting rate of 97% (i.e.134 out of 138 RRFs were submitted), yet only 16 (69.4%) of the facilities’ timely submitted their reports. Of the total reports submitted to the next higher level, 135 (97.8%) of them were found to be complete (Fig. 5).

#### Bin-card data accuracy

**Fig. 6:**
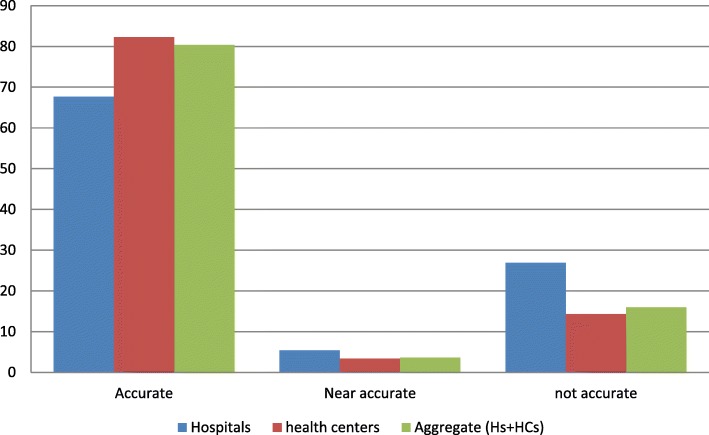
The accuracy of bin-cards by facility type and the overall performance of the selected public health facilities in East Wollega zone, May30/2017

A physical count was conducted to determine if a discrepancy exists between a physical stock on hand in the storeroom and ending balance on bin-cards. Accordingly, of the total sampled bin-cards, 647 (80.36%) of the records were found to be accurate, and 129 (16%) were inaccurate. However, the health centers had recorded 576 (82.3%) of their bin-cards accurately, and the hospitals had 71 (67.7%) accurate & 28 (27%) inaccurate bin-cards (Fig. 6).

A chi-square test was done to determine if an association exists between bin-card data accuracy and possible factors. Consequently, a significant association was observed between bin-card data quality and supervision, *X*^*2*^ (2, *N* = 805) =7.92, *P* = 0.019 & type of profession, *X*^*2*^ (2, *N* = 805) =6.39, *P* = 0.041 (Table 4).

**Table 4:** The association of bin-card data accuracy, and possible contributing factors in the selected public health facilities of East Wollega zone, May30/2017

Variables	Bin-card data accuracy		
	Pearson chi-square		
	Value	Df*	P-values
Service year	5.619	2	0.06
Training	1.558	2	0.459
Feedback	5.187	2	0.075
Supervision	7.918	2	0.019
Type of profession	6.394	2	0.041

* degree of freedom

### Qualitative results

Face to face in-depth interview was conducted to explore the challenges associated with LMIS performance.The participants were selected based on their service experience in pharmaceutical logistics management. Accordingly, six storekeepers and five pharmacy heads with service year of greater than five were involved. The key informants identified various types of problems, and the findings were then thematically analyzed by categorizing based on the feature of the data.

#### Human resource and staff’s commitment related challenges

##### Human resource-related challenges

Maintaining quality pharmacy services requires adequate skilled human resources. However, the number of pharmacy professionals in the selected public health facilities was not adequate. The main reasons mentioned by the interviewees were high staff attrition rate, few pharmacy professionals in the market, and the government and private higher institutions are producing a limited number of pharmacy professionals. One of the pharmacy heads explained the problem as follows,

*“Since the majority of the pharmacy professionals in Ethiopia are not satisfied with their current profession (because of salary and lack of recognition from the government), a significant number of pharmacists and pharmacy technicians are shifting to other business fields. Unless the government timely solves the issue, for instance, through promoting private colleges to produce at least diploma graduates, and also fulfill the needs of the pharmacists as much as possible, the problem will get more burdensome in the future. Currently, we use the available staffs more to dispense drugs than managing logistics information.”*

The study also explored that the shortage of pharmacists and pharmacy technicians levied burdens on the available staff. In the majority of the study facilities, a single person had multiple responsibilities. For instance, an individual assigned to work on LMIS also had responsibilities of carrying out other pharmacy activities. One of the key informants (KI) reported that,

*“Currently, in my facility, I have a responsibility of dispensing drugs, managing store and preparing LMIS reports. Thus, I could not get sufficient time to update the logistics records. I have recommended the facilities to recruit additional staffs.”*

##### Staffs’ commitment related challenges

Most of the interviewees, especially the pharmacy heads mentioned that lack of staff commitment was one of the challenges influencing the pharmacy activities. The problem was even more severe among the staffs in the rural health facilities. The key informants mentioned some of the reasons behind the issue. For example, poor infrastructures, job dissatisfaction, lack of technologies, and inadequate incentives particularly a limited opportunity for further education discouraged the workers not to execute their tasks properly. One of the pharmacy head tried to elucidate the situation as follows,

*“When we recommend the staffs working in dispensing units to record and report logistics data properly, they feel discomfort and prefer to resign their job. Their main reasons are inadequate incentives, lack of electronic LMIS devices, and electric power interruptions.”*

#### Managerial related problems

##### Poor supportive supervision and feedback

The facilities were also facing poor management support to manage their information systems effectively. In the majority of the study facilities, the top-level managers were not considering LMIS as a basic pharmacy service. As a result, the staff lacked the inspiration to manage logistics data appropriately, and therefore, the majority of them relied on guesswork. They stated that weak supportive supervision and poor constructive feedbacks on LMIS management led to unreliable data quality. One of the interviewees reported that,

*“I believe that supportive supervision has a profound impact in filling knowledge gaps and also encouraging workers. However, in our facility, there is irregular supervision with no feedback. Some of the supervisors are negligent. They usually visit the facility with no comment and back to their office.”*

##### Poor communication between the health facility managers and suppliers

For better decision-making, the facilities and the corresponding suppliers should have a regular communication on the current logistics management practice and challenges in the health facilities and at supplier point. Otherwise, the decisions that do not involve both parties will discourage the lower level staffs to maintain quality information. In the current study, poor communication between the health facilities and pharmaceutical fund and supply agency (PFSA) was among the challenges that affect the logistics management information systems.

This can be exemplified by one of a KI as follows:

*“Urging the facilities to prepare & send RRF regularly is one strong side of PFSA to enhance reporting rates. However, on the other hand, we request the PFSA a double quantity of the actual demand, since it commonly provides less than the required amount. Such practice discourages us to submit a quality report.”*

## Discussions

Availability of standard logistics recording and reporting tools/formats and proper use of these tools have a substantial role in the implementation of effective and efficient logistics management information systems [22]. In the present study, almost all types of the recording and reporting formats were 100% available and entirely used in the facilities to record and report logistics data. This is in line with the study done in East Shewa zone on inventory management performance [23] and the study conducted in Addis Ababa on logistics management information system of laboratory commodities [24], in which the study facilities had the required logistics recording and reporting tools or formats and use them to capture essential logistics data. However, the majority of the facility in a current study, particularly health centers (70% of health centers) were relying on paper-based activities, only 2/3 the hospitals were using automated recording systems. The result is similar to the study conducted in Kenya on the utilization of healthcare information, wherein the facilities mainly depend on a paper-based reporting system and poorly supported by an automated system [25]. Nevertheless, electronic recording and reporting system enhances the logistics management information system (LMIS) performances through reducing errors & task burden, saving time, and improving reporting rates [26]. The poor utilization of electronic LMIS might be because of frequent power interruption, inadequate skilled human resources and the geographical location of the health facilities where electric power was completely unavailable.

Regarding data quality of reports, it has substantial impacts on the quality of health care and even on government budgets for the maintenance of health services. Therefore, every facility in a supply chain needs to improve its data quality and timely share it to maintain health care at an optimal level [27]. In the current study, of the total sampled RRFs, 64.6% were accurate, 69.4% were timely reported and 97.8% of the reports were found to be complete. These indicate the facilities’ weak performance in recording their logistics data and reporting to the next higher level. The findings are slightly lower than the assessment conducted by SIAPS in Cameroon and Burundi, wherein the accuracy and timeliness of their reports were 75% & 90%, respectively [28]. The reason might be a difference in the degree of commitments by higher officials in providing regular supportive supervision and feedback on the facilities performances and a shortage of skilled human resources. Factors including staff training, availability of an electronic recording system, and the educational level of staffs might also be significantly affecting the data quality of LMIS in the present study.

Regarding a report submission rate, the health facilities of the current study submitted 97% of the expected reports to the pharmaceutical fund and supply agency of Ethiopia (PFSA). This is higher than that of the study done in Malawi and Nigeria, where the reporting rates were 58% & 12.3%, respectively [6, 29]. The difference might be due to the urging principle of PFSA, i.e., forced ordering system. Such system pushes the facility to prepare and submit their reports bi-monthly to timely forecast the facilities need (“no report no drug principle”).

The inventory record accuracy is one of the components of data quality that has a paramount impact on a report data accuracy. The present study revealed that from the total of reviewed bin-cards in the selected health facilities, 79.1% were accurately recorded (had similar data with physical count). It is higher compared to previous studies including the study conducted in Addis Ababa on a logistics management information system for laboratory commodities & South Sudan on pharmaceutical logistics management, where the facilities in both studies had inventory record accuracy of 38.9% & 27%, respectively [24, 26]. The difference might be because of the number and types of facilities selected for the studies and the size of the study area. For instance, the study done in South Sudan covered four geographical sites (four states) and private pharmacies In the same fashion, the study conducted in Addis Ababa included a higher number of facilities (i.e.43 health facilities) compared to the current study, where only 23 facilities evaluated. As limitations, because of budget constraints, the present study was limited to the evaluation of commonly and consistently used documents in the health facilities for decision-making. The study also did not include health posts. Because, in Ethiopia, health posts manage only a few program drugs like family planning and other medicines for minor ailments. It would be good if the study evaluated an information system at the supplier points too. Therefore, the future studies can involve both health facilities and immediate suppliers to investigate the management information system at both sides and also explore the possible challenges the facilities and suppliers are experiencing.

## Conclusions

From this study, we concluded that the facility report quality and inventory record accuracy require improvements, nonetheless, their reporting rate was encouraging. The major challenges that influence the facilities to manage their information system appropriately were identified to be a shortage of pharmacy professionals, lack of commitment from top-level management and inability to implement automated recording and reporting system. The study also identified staffs that have taken integrated pharmaceutical logistics system training yet not contributed to a logistics management information system and even not volunteers to do so. Their main reasons were the burden of tasks and absence of an automated recording system in the facilities. Generally, factors like training of staffs, availability of automated record systems, supportive supervision and feedback report had a significant association with the RRF data accuracy. Therefore, pharmaceutical fund and supply agency and partners should increase their frequency of supportive supervision and also provide constructive feedback to the facilities. The facilities, especially the health centers together with the concerned stakeholders, should strengthen their LMIS by implementing automated recording systems.

## Abbreviations

CI: Confidence of interval
e-LMIS: Electronic logistics management information system
IFRR: Internal facility report & requisition form
IPLS: Integrated pharmaceutical logistics system
LIAT: Logistics indicator assessment tool
MCH & FP: Maternal, and child health & family planning
PFSA: Pharmaceutical fund and supply agency
RRF: Report and requisition form
USAID: United states agency for international development
